# Pharmacokinetics, Absolute Bioavailability, and Nonlinear Topical Absorption of a Fluralaner–Moxidectin Spot-On Formulation in Cats

**DOI:** 10.3390/vetsci13070700

**Published:** 2026-07-17

**Authors:** Jinyan Meng, Qinyao Wu, Runlin Yu, Zeyu Wen, Sumeng Chen, Yang Zhang, Nuoyu Xu, Shuyan Guo, Xilu Sun, Xingyuan Cao

**Affiliations:** 1 Department of Veterinary Pharmacology and Toxicology, College of Veterinary Medicine, China Agricultural University, Beijing 100193, China; jinyanmeng00@outlook.com (J.M.); wuqinyao7711@163.com (Q.W.); yurunliny@163.com (R.Y.); wenzy1124@163.com (Z.W.); phdchensumeng@163.com (S.C.); zhangyang981018@163.com (Y.Z.); 2019305010115@cau.edu.cn (N.X.); guoshuyan1566@163.com (S.G.); 18796119112@163.com (X.S.); 2 Key Laboratory of Detection for Veterinary Drug Residues and Illegal Additives, Ministry of Agriculture and Rural Affairs of the People’s Republic of China, Beijing 100193, China

**Keywords:** fluralaner–moxidectin, cats, spot-on, nonlinear pharmacokinetics, drug interaction

## Abstract

Long-acting spot-on formulations are widely used for parasite control in cats, yet the pharmacokinetics of combined fluralaner and moxidectin remain incompletely defined beyond the recommended dose. This study evaluated the pharmacokinetics, absolute bioavailability, and dose proportionality of a novel fixed-combination spot-on formulation in healthy cats after topical administration of fluralaner/moxidectin at 40/2, 80/4, and 120/6 mg/kg BW, and compared intravenous administration of each compound alone or in combination. Forty-eight healthy cats were allocated to six groups of eight cats each. Non-compartmental analysis and a power model were used to characterize systemic exposure and dose proportionality. After intravenous co-administration, fluralaner exposure increased whereas moxidectin exposure decreased compared with single-agent administration, indicating a potential pharmacokinetic interaction. Topical administration produced slow absorption and sustained systemic persistence, with T_max_ values occurring approximately 10–18 days after dosing and quantifiable concentrations maintained throughout the 160 days sampling period. Systemic exposure increased non-proportionally across the tested dose range, and absolute bioavailability was limited and dose-dependent, ranging from 9.7% to 26.9% for fluralaner and from 10.22% to 21.28% for moxidectin. No adverse reactions were observed. These findings demonstrate nonlinear topical absorption, and prolonged systemic persistence, supporting further dose optimization and formulation development of this long-acting feline antiparasitic delivery system.

## Simple Summary

Long-acting spot-on products are widely used for parasite control in cats, but their drug absorption after skin application can be complex. This study evaluated a new fixed-combination spot-on formulation containing fluralaner and moxidectin in healthy cats at three topical dose levels, with intravenous treatments used for comparison. After topical administration, both drugs were absorbed slowly, reached peak blood concentrations about 10 to 18 days after treatment, and remained measurable throughout the 160-day study period. Blood exposure did not increase in a simply dose-proportional manner, suggesting that topical absorption was prolonged and variable. The intravenous comparison also indicated a moderate pharmacokinetic interaction between the two compounds when given together. No adverse reactions were observed. These findings suggest that dose selection and formulation development for this feline spot-on product should rely on pharmacokinetic evidence rather than simple linear dose scaling.

## 1. Introduction

With economic and social development, demand for companion animals such as dogs and cats is increasing. However, parasitic diseases in pets have become one of the most common health issues in companion animal practice [1,2]. Many companion-animal parasites are zoonotic; therefore, parasitic diseases not only endanger companion animals but also threaten public health, underscoring the urgent need for effective prevention and control [3,4,5,6]. Globally, the infection rates of parasites in dogs and cats remain high. For example, a survey by Riggio et al. [7] in central Italy showed that approximately 31% of 239 domestic dogs were infected with at least one intestinal or pulmonary parasite, and about 35% of 81 domestic cats tested positive for at least one parasite, with a relatively high proportion of zoonotic *Toxocara* species. Single-component anthelmintics typically have a limited spectrum of activity and rarely cover nematodes, cestodes, and ectoparasites simultaneously. In contrast, multidrug combinations often require cumbersome administration schedules, increasing the risk of treatment refusal in pets and poor owner adherence [8,9]. Through rational formulation, fixed-dose products can provide synergistic, multitarget efficacy against common endo- and ectoparasites in a single dose, thereby improving treatment compliance [10]. Several studies have demonstrated that topical feline endectoparasiticide combinations can provide broad-spectrum efficacy against common parasites of cats. For instance, NexGard^®^ Combo, a topical formulation containing esafoxolaner, eprinomectin, and praziquantel, was developed for cats with, or at risk of, mixed infestations involving ectoparasites, gastrointestinal helminths, cardiopulmonary worms, and cestodes [11]. Controlled studies further showed high efficacy of this combination against major feline endoparasites, including *Toxocara* cati and Dipylidium caninum, as well as against *Ctenocephalides felis* fleas [12,13]. Similarly, the fipronil/(S)-methoprene/eprinomectin/praziquantel combination product has demonstrated high efficacy and safety against nematode and cestode infections in domestic cats under field conditions [14]. As a broad-spectrum parasiticide, BROADLINE^®^ spot-on solution (containing fipronil, methoprene, eprinomectin, and praziquantel) has demonstrated good preventive and therapeutic efficacy against roundworms, tapeworms, and fleas in cats [15]. Therefore, the rational use of combination products represents a key strategy for improving the prevention and control of parasites in pets.

The fluralaner–moxidectin combination spot-on is a notable innovation in this field. Fluralaner is an isoxazoline-class insecticide. As demonstrated by Gassel et al., this class of compounds selectively antagonizes γ-aminobutyric acid (GABA)-gated and L-glutamate-gated chloride channels in the arthropod nervous system, thereby disrupting inhibitory neurotransmission and ultimately causing nervous system dysfunction and death [16]. Isoxazolines exhibit extremely low sensitivity to mammalian GABA receptors, and mammals lack glutamate-gated chloride channels (GluCls), which accounts for their favorable safety profile [17]. The two components act through distinct mechanisms to provide complementary coverage against a broad spectrum of internal and external parasites. Fluralaner is highly effective against ectoparasites such as fleas, ticks, and ear mites (*Otodectes cynotis*), whereas moxidectin is indicated for heartworm prevention and the treatment of intestinal nematodes, including roundworms and hookworms.

After topical spot-on administration, systemic exposure is governed by a sequence of formulation- and host-dependent processes, including drug release from the vehicle, spreading over the skin surface, partitioning into the stratum corneum and follicular structures, passage across the cutaneous barrier, and subsequent systemic disposition [18,19,20]. These processes are influenced by the physicochemical properties of the active compound, including lipophilicity, molecular size, and ionization state, as well as by formulation composition, application volume, skin condition, hair coat characteristics, and post-application grooming behavior [21]. For fixed-combination products administered according to body-weight bands, cats at different positions within the same band may receive different mg/kg doses; therefore, proportional increases in AUC and Cmax cannot be assumed without formal dose-ranging pharmacokinetic evaluation [22,23]. Moreover, intravenous reference data are required to distinguish absorption-related changes in systemic exposure from changes in systemic clearance and to estimate the absolute bioavailability of the topical formulation [24].

Previous studies have demonstrated the clinical efficacy and safety of fluralaner–moxidectin spot-on formulations for the control of parasitic infections in cats. However, the dose-dependent pharmacokinetic behavior of this fixed combination, particularly the relationship between topical dose and systemic exposure, remains insufficiently defined. This study aimed to characterize the pharmacokinetics of fluralaner and moxidectin after separate and combined intravenous administration, to evaluate potential pharmacokinetic interactions between the two compounds, and to provide an intravenous reference for the estimation of absolute bioavailability. In healthy cats, systemic exposure (AUC and C_max_) of fluralaner and moxidectin increased non-proportionally with dose following topical application of the fixed-combination spot-on formulation at 40/2, 80/4, and 120/6 mg/kg body weight. This was formally evaluated using a power model, with the slope coefficient (β) and its confidence interval as the primary metrics. The study also included intravenous groups receiving the combination or the single compounds, to determine the absolute bioavailability of the topical formulation and to characterize the absorption and elimination profiles of the drugs in cats. This study design enabled us to distinguish absorption-driven changes in systemic exposure from changes in terminal elimination and to determine whether exposure after topical administration could be predicted by simple dose scaling. These findings provide a pharmacokinetic rationale for dose optimization, formulation development, and rational clinical use of fluralaner–moxidectin spot-on formulations in cats, particularly in the context of body-weight band dosing and the potential limitations of extrapolating exposure across dose ranges. More broadly, this work may support improved long-acting antiparasitic strategies in companion animals and contribute to parasite control programs with potential relevance to zoonotic risk reduction.

## 2. Materials and Methods

### 2.1. Chemicals and Reagents

Acetonitrile (ACN) and formic acid (FA) were procured from Thermo Fisher Scientific (Macquarie Park, NSW, Australia). Ultrapure water was produced using a Milli-Q system (Merck Millipore, Burlington, MA, USA). Fluralaner (purity 99.9%) was obtained from Dr. Ehrenstorfer, moxidectin (purity 94.1%) from the European Pharmacopoeia (Ph. Eur.) (Strasbourg, France), fluralaner-D_4_ (purity 99.7%) from Witega (Berlin, Germany), and moxidectin-D_3_ (purity 95.7%) from TLC Pharmaceutical Standards. The fluralaner–moxidectin combination product was obtained from Merck (Rahway, NJ, USA).

### 2.2. Experimental Animals

Because this was an exploratory pharmacokinetic study rather than a clinical efficacy trial, no formal a priori power analysis was performed. The sample size was selected based on published feline PK studies using comparable dose-proportionality or dose-related PK designs, feasibility of intensive serial sampling, and animal welfare considerations. A total of 48 healthy British Shorthair cats, comprising 24 males and 24 females, were obtained from Beijing Anmosai Biotechnology Co., Ltd (Beijing, China). All cats were acclimated to controlled housing conditions for a two-week period before the experiment. Cats were included if they were clinically healthy adult cats, with body weight within 2.8–3.6 kg, normal findings on physical examination, and no clinically relevant abnormalities in hematology and serum biochemistry before drug administration. Cats were excluded if they had evidence of systemic disease, abnormal baseline laboratory findings, pregnancy or lactation, recent administration of drugs that could affect pharmacokinetics, poor compliance with dosing or blood sampling, or any adverse clinical condition judged by the veterinarian to compromise welfare or data interpretation. Animals showing any such signs were excluded from the study; those without evidence of illness were enrolled. This study received approval from the Laboratory Animal Welfare and Animal Experimental Ethical Committee of China Agricultural University (Beijing, China; approval No. AW72406202-2-01).

### 2.3. Drug Administration and Study Design

The experimental cats were randomly assigned to six groups, with eight cats per group. The grouping and corresponding treatments are summarized in Table 1.

Approximately 1 mL of whole blood was collected prior to administration (0 h) and at 15 min, 2 h, 4 h, 8 h, 12 h, and 1, 3, 5, 7, 10, 14, 21, 28, 35, 42, 49, 56, 63, 70, 77, 84, 98, 115, 130, 145, and 160 days post-intravenous administration. For transdermal administration, plasma samples were collected at the same time points, except that the first sample was obtained 8 h post-dose. All sampling procedures and total blood volumes collected were conducted under veterinary supervision. Blood samples were centrifuged at 4000 rpm for 10 min, and the resulting plasma was harvested and stored at −20 °C until analysis.

### 2.4. Instrument Conditions and Sample Preparation

Chromatographic separation was performed using an Agilent 1290 Infinity II UPLC (Agilent Technologies, Santa Clara, CA, USA) system equipped with a Phenomenex Kinetex C18 column (2.1 × 50 mm, 2.6 μm) (Phenomenex, Torrance, CA, USA). The mobile phases consisted of 2 mM ammonium acetate (A) and acetonitrile (B), delivered at a flow rate of 0.3 mL/min. The elution gradient was as follows: 0~3 min, 80% A to 0% A; 3~5.5 min, 0% A; 5.5~6 min, 0% A to 80% A; 6~8 min, 80% A. The column temperature was maintained at 40 °C. The source conditions were as follows: source temperature, 300 °C; desolvation temperature, 300 °C; capillary voltage, 3.0 kV; desolvation gas flow, 300 L/h [25].

A 100 μL aliquot of plasma was mixed with 10 μL of internal standard solution (fluralaner-D_4_, 10,000 ng/mL; moxidectin-D_3_, 1000 ng/mL) in a 1.5-mL microcentrifuge tube. After vortexing, 900 μL of acetonitrile was added. The tube was shaken for 5 min, then centrifuged at 14,000 rpm at 4 °C for 10 min. The supernatant was filtered through a 0.22 μm membrane, transferred to a vial, and 5 μL was injected for UPLC-MS/MS (Agilent Technologies, Santa Clara, CA, USA) analysis. This simple and rapid method enables the first simultaneous quantification of fluralaner and moxidectin in a combined formulation.

### 2.5. Method Validation

Calibration curves for fluralaner and moxidectin were prepared over three consecutive days within linear ranges of 5.0–2500.0 ng/mL and 0.5–250.0 ng/mL, respectively, and linearity was assessed. Selectivity was evaluated by processing six blank plasma samples obtained from different cats using the previously described sample preparation procedure. Carryover was assessed by injecting duplicate blank samples immediately following each injection of the upper limit of quantitation (ULOQ) standard. Quality control (QC) samples were prepared at four concentration levels: above the lower limit of quantification (LLOQ, 5.0/0.5 ng/mL), low QC (LQC, 15.0/1.5 ng/mL), medium QC (MQC, 800.0/80.0 ng/mL), and high QC (HQC, 2000.0/200.0 ng/mL). Method accuracy and precision were validated over three consecutive days. Recovery was assessed by processing and injecting QC samples at LQC, MQC, and HQC levels, and calculating the recoveries of fluralaner, moxidectin, and the internal standards. Dilution integrity was evaluated by diluting cat plasma samples containing fluralaner and moxidectin with blank plasma via one 10-fold and three serial 10-fold dilutions, followed by processing and analysis. Stability studies were conducted using QC samples at low (15.0/1.5 ng/mL), medium (800.0/80.0 ng/mL), and high (2000.0/200.0 ng/mL) concentrations. The following stability conditions were evaluated: room temperature, freeze–thaw, post-preparative, and long-term stability after storage at −20 °C for 112 days. In addition, the matrix effect of the method was assessed.

### 2.6. Data and Statistical Analysis

Fluralaner and moxidectin concentrations in blood were quantified using the validated UPLC-MS/MS method. Pharmacokinetic parameters were calculated using non-compartmental analysis (NCA) in WinNonlin software (version 8.3.4; Certara, Pharsight, Mountain View, CA, USA) and presented as mean ± standard deviation (SD). A linear mixed-effects power model was fitted to log-transformed AUC_0−t_, with log dose as a fixed effect and animal as a random effect. The slope coefficient (β) and its 95% confidence interval were used to evaluate whether systemic exposure increased in proportion to dose. This model makes full use of repeated-measures data and represents the current standard for evaluating dose proportionality. Drug concentration–time profiles were plotted using Origin 2024. For key PK parameters such as clearance (CL), volume of distribution (Vd), and AUC of fluralaner and moxidectin between the single-agent and combination intravenous groups, an independent-samples *t*-test was applied. A *p*-value of <0.05 was considered statistically significant.

## 3. Results

### 3.1. Intravenous Pharmacokinetics of Fluralaner and Moxidectin: Single-Agent and Combination Administration

**Figure 1:**
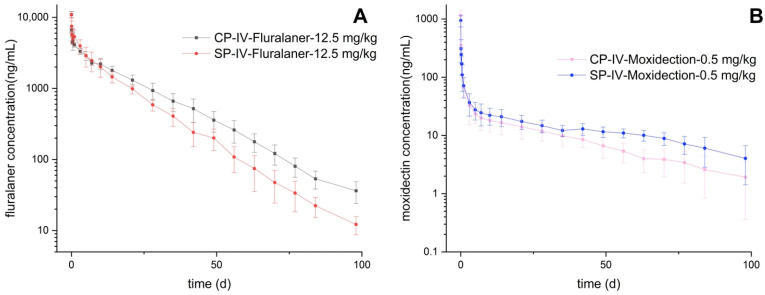
Semilogarithmic plasma concentration–time profiles of fluralaner and moxidectin following single intravenous administration (SP) or combined administration (CP). Note: (**A**) Fluralaner; (**B**) Moxidectin. SP, single preparation; CP, combination preparation. The same abbreviations are used hereinafter.

Mean plasma concentration–time profiles of fluralaner and moxidectin following a single intravenous bolus of either the individual agent (fluralaner 12.5 mg/kg or moxidectin 0.5 mg/kg) or the combination (fluralaner 12.5 mg/kg + moxidectin 0.5 mg/kg) in cats are presented in Figure 1, with the corresponding non-compartmental pharmacokinetic parameters summarized in Table 2.The statistical comparison of the key pharmacokinetic parameters is presented in Table 3.

For fluralaner, AUC_0−t_ following single-drug administration (65,292.74 ± 8726.43 ng·d/mL) was significantly lower than that observed after combination administration (77,527.95 ± 10,539.80 ng·d/mL; *p* = 0.024). Total plasma clearance (CL) of fluralaner was 193.96 ± 24.81 mL/d/kg when administered alone and 163.48 ± 22.62 mL/d/kg when co-administered with moxidectin (*p* = 0.022). Similarly, the volume of distribution at steady state (Vz) and terminal elimination half-life (t_1/2_) were comparable between the two groups (Vz: 3339.60 ± 635.53 vs. 3524.97 ± 519.07 mL/kg; t_1/2_: 11.86 ± 1.19 d vs. 14.99 ± 1.32 d). For moxidectin, AUC_0−t_ values were 1584.55 ± 428.54 ng·d/mL for the single agent and 1137.92 ± 302.35 ng·d/mL for the combination (*p* = 0.030). CL, V, and t_1/2_ of moxidectin were 332.18 ± 105.80 mL/d/kg, 14,480.62 ± 4465.34 mL/kg, and 31.02 ± 8.44 d after single dosing, and 472.58 ± 196.42 mL/d/kg, 17,499.73 ± 8140.55 mL/kg, and 25.60 ± 6.38 d after combination administration, respectively.

### 3.2. Dose-Dependent Transdermal Pharmacokinetics of the Fluralaner–Moxidectin Combination Spot-On Formulation

**Figure 2:**
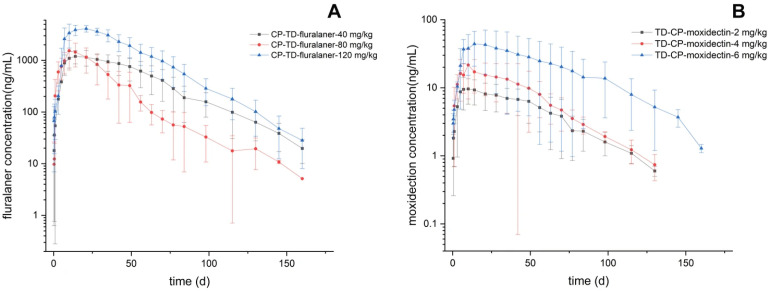
Semilogarithmic plasma concentration–time profiles of fluralaner and moxidectin following topical application of the combination spot-on formulation at fluralaner: 40 mg/kg/moxidectin: 2 mg/kg, fluralaner: 80 mg/kg/moxidectin: 4 mg/kg, and fluralaner: 120 mg/kg/moxidectin: 6 mg/kg of the recommended dose. Note: (**A**) Fluralaner; (**B**) Moxidectin.

The combination spot-on formulation containing fluralaner and moxidectin was applied to the shaved dorsal skin of cats at three escalating doses: low (fluralaner 40 mg/kg, moxidectin 2 mg/kg), medium (fluralaner 80 mg/kg, moxidectin 4 mg/kg), and high (fluralaner 120 mg/kg, moxidectin 6 mg/kg). Plasma samples were collected over a 160-day period. Plasma concentration-time profiles for both drugs are presented in Figure 2, with the main transdermal pharmacokinetic parameters summarized in Table 4 and Table 5.

Pharmacokinetic parameters of fluralaner following single transdermal administration in cats are summarized in Table 4. Systemic exposure exhibited a pronounced non-monotonic relationship with dose: mean AUC_0−t_ was 66,739.99 ± 26,844.99 ng·d/mL at 40 mg/kg, decreased unexpectedly to 48,235.34 ± 18,913.22 ng·d/mL at 80 mg/kg, and subsequently increased to 192,651.15 ± 39,748.55 ng·d/mL at 120 mg/kg. Accordingly, dose-normalized AUC declined 1.4-fold from 40 mg/kg to 80 mg/kg and increased 4.0-fold from 80 mg/kg to 120 mg/kg, clearly indicating deviation from dose proportionality. This biphasic pattern, together with moderate variability, suggests it is not an artifact and implicates a dose-dependent interplay between drug permeation and stratum corneum barrier function at the application site. C_max_ increased supraproportionally: the 2.5-fold rise from 40 mg/kg to 120 mg/kg markedly exceeded the 1.4-fold rise from 40 mg/kg to 80 mg/kg, confirming that facilitation of transdermal flux predominates at the highest dose. In contrast, elimination remained linear, as indicated by a dose-independent terminal half-life (14.2–20.3 days) and an extrapolated AUC of less than 1%. The prolonged T_max_ at 40 mg/kg and 120 mg/kg (16.9 and 18.3 days) and extended MRT_last_ (>37 days) suggest rate-limited absorption or formation of a cutaneous depot. Collectively, transdermal fluralaner exhibited a complex nonlinear pharmacokinetic profile, consistent with dose-dependent modulation of the stratum corneum barrier-partially compromised at lower doses and actively facilitated at the highest dose, resulting in a supraproportional increase in systemic exposure.

Pharmacokinetic parameters of moxidectin following single transdermal administration in cats are summarized in Table 5. Systemic exposure increased non-proportionally with dose: mean AUC_0−t_ values were 534.30 ± 242.69, 930.02 ± 460.55 and 2906.08 ± 2099.25 ng·d/mL at 2 mg/kg, 4 mg/kg, and 6 mg/kg, respectively. Dose-normalized AUC decreased by 13% from 2 mg/kg to 4 mg/kg and then increased 2.04-fold from 4 mg/kg to 6 mg/kg, revealing a biphasic deviation from dose proportionality, shifting from subproportional to supraproportional. C_max_ similarly increased 1.9-fold (2 mg/kg to 4 mg/kg) and 2.2-fold (4 mg/kg to 6 mg/kg). Elimination was linear, with a dose-independent terminal half-life (23.1–27.5 days; λz ≈ 0.03 day^−1^) and a low extrapolated AUC (≤6.7%). Mean T_max_ values were 15.63, 10.50, and 13.75 days for the three doses; variability in T_max_ was greatest at 2 mg/kg, indicating erratic absorption at the lowest dose. MRT_last_ was comparable between 2 mg/kg and 4 mg/kg (36.50 and 35.26 days) but prolonged at 6 mg/kg (44.68 days), consistent with rate-limited absorption or formation of a cutaneous depot at the highest dose. In summary, transdermal moxidectin provided sustained exposure characterized by dose-dependent, biphasic nonlinear absorption, while elimination remained linear.

### 3.3. Absolute Bioavailability and Dose Proportionality

Using the AUC_0−t_ obtained from the intravenous combination injection as the reference, the absolute bioavailability (F%) of the combination spot-on formulation was calculated for each dose. For fluralaner, F% values were 26.90%, 9.72%, and 25.88% at the low, medium, and high doses, respectively. For moxidectin, the corresponding F% values were 11.74%, 10.22%, and 21.28% across the three dose groups.

**Figure 3:**
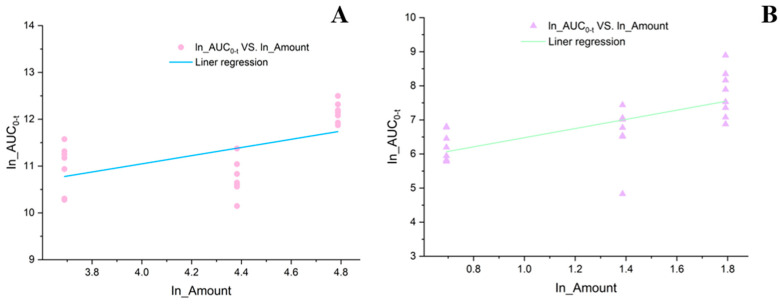
Dose-AUC linearity of fluralaner and moxidectin. Note: (**A**) Fluralaner; (**B**) Moxidectin.

Figure 3 and Table 6 illustrate the dose-AUC_0−t_ relationships for fluralaner and moxidectin. A linear mixed-effects model (log AUC_0−t_ ~ log dose, random effect: animal) was employed to assess dose proportionality using a power function (Equation (1)). Dose proportionality would be concluded if the 95% confidence interval (CI) for β fell entirely within the acceptance interval (Substituting the relevant parameters of this study into Equation (2) yields an acceptance interval of 0.80–1.20). The 95% CIs for β were 0.31–1.44 (fluralaner) and 0.70–1.99 (moxidectin), both exceeding the prespecified limits. Therefore, dose proportionality could not be established within the evaluated dose range and statistical power of the present study.

(1) Ln(Parameter) = α + β × Ln(Dose)

(2) $$\left[1+\frac{Ln(QL)}{Ln(r)},\;1+\frac{Ln(QU)}{Ln(r)}\right]$$

r: Ratio of the highest dose to the lowest dose.

QL: Lower equivalence limit-0.8 (for AUC).

QU: Upper equivalence limit-1.25 (for AUC).

**Table 6:** Results of linearity analysis for fluralaner and moxidectin following transdermal administration of the combined formulation, based on a linear mixed-effects model.

Power Model	Slope Estimate	*p* Value	Lower CI 95%	Upper CI 95%
Fluralaner AUC	0.87	0.004	0.31	1.44
Moxidectin AUC	1.35	0.000	0.70	1.99

## 4. Discussion

The present study provides an integrated pharmacokinetic evaluation of fluralaner and moxidectin in cats after intravenous administration and after topical administration of an escalating-dose fixed-combination formulation. Previous feline pharmacokinetic work has shown that fluralaner persists for an extended period after both topical and intravenous administration in cats [26]. The extended ectoparasiticidal activity of fluralaner is consistent with its selective inhibition of arthropod γ-aminobutyric acid- and glutamate-gated chloride channels [16]. Moxidectin is a macrocyclic lactone with broad endectocidal activity and pharmacokinetic behavior characterized by high lipophilicity, extensive distribution, and prolonged persistence [27]. The present study extends this background by addressing two less well-defined questions: whether fluralaner and moxidectin influence each other after systemic co-administration, and whether topical exposure increases proportionally across the investigated dose range.

Following intravenous administration, co-administration with moxidectin significantly increased systemic exposure to fluralaner, as indicated by a higher AUC_0−t_ and lower plasma clearance compared with fluralaner alone. The terminal half-life and distribution-related parameters of fluralaner were not markedly changed, suggesting that the interaction was not primarily driven by a major alteration in the terminal elimination phase. This interpretation is consistent with previous observations that fluralaner has slow elimination and long systemic persistence in cats [26]. Therefore, the intravenous data suggest that combination administration may modestly influence the systemic disposition of fluralaner, possibly through effects on early distribution, plasma–tissue partitioning, or binding-related processes. Because plasma protein binding, tissue distribution, transporter activity, and excretion were not measured, the mechanistic basis of this interaction remains to be defined in future studies.

For moxidectin, systemic exposure was lower after intravenous combination administration than after single-agent administration, whereas clearance was directionally higher in the combination group. Macrocyclic lactones can show disposition patterns influenced by tissue distribution and transporter-mediated efflux, particularly P-glycoprotein-related processes [28]. Moxidectin has also been reported to differ from some other macrocyclic lactones in its tissue distribution and persistence, which may contribute to compound-specific interaction patterns [29]. The opposite directions of the interaction observed for fluralaner and moxidectin suggest that these two active ingredients should not be assumed to behave as pharmacokinetically neutral co-components. Because the terminal slopes were broadly comparable between single-agent and combination administration, these findings are best interpreted as a moderate disposition-level interaction rather than a profound change in intrinsic elimination. Variability in early distribution, plasma or tissue binding, and transporter-related disposition may also contribute to the different interaction patterns observed for the two analytes.

After topical administration, both active ingredients showed sustained systemic exposure over several weeks. This sustained exposure is consistent with the long-acting clinical profile of fluralaner–moxidectin spot-on products in cats. A European field study reported that a single topical application of fluralaner plus moxidectin was well tolerated and effective against fleas and ticks in naturally infested cats for 12 weeks [30]. Another multicenter field study showed that the same combination provided broad-spectrum treatment of gastrointestinal nematode infections in cats [31]. Experimental studies have also demonstrated efficacy of fluralaner plus moxidectin against *Otodectes cynotis* infestations in cats [32]. More recent studies have extended the evidence base to prevention of feline aelurostrongylosis after topical administration of the same fixed combination [33]. The present study adds a pharmacokinetic explanation for these long-duration outcomes by showing persistent systemic exposure to both compounds after topical dosing.

The most important finding of the present study is that systemic exposure did not increase in a dose-proportional manner. For fluralaner, AUC_0−∞_ decreased unexpectedly from the low-dose group to the medium-dose group and then increased markedly at the high dose. For moxidectin, dose-normalized exposure showed a similar pattern. In the power-model analysis, the slope estimate for fluralaner was close to proportionality, whereas the confidence interval was wide; for moxidectin, the confidence interval included 1.0. These results indicate that dose proportionality was not established within the evaluated dose range and study power. The analysis therefore describes exposure scaling and systemic availability, rather than pharmacodynamic potency or concentration–effect relationships. The stratum corneum is the major barrier to percutaneous drug absorption, and changes in this barrier can substantially alter drug permeation [34]. Topical drug delivery is also influenced by vehicle composition, drug thermodynamic activity, skin hydration, and formulation-dependent barrier modulation [35]. Accordingly, the observed dose–exposure pattern is compatible with variation in topical input, although systemic disposition and inter-individual variability may also contribute.

The lack of clear dose dependence in terminal half-life further supports an absorption-driven mechanism. Across the investigated topical dose range, terminal half-life and λz were relatively stable for both compounds, whereas AUC and C_max_ changed non-proportionally. Long-acting transdermal formulations often depend on formulation-controlled release, depot behavior, and gradual diffusion across the skin rather than on changes in systemic clearance [36]. The prolonged T_max_ and extended MRT_last_ observed in the present study, especially at the highest dose, are consistent with rate-limited input from a cutaneous or subcutaneous reservoir. Recent reviews of transdermal delivery also emphasize that skin retention and delayed redistribution can contribute to sustained systemic exposure [37]. Thus, the topical combination appears to show formulation-dependent cutaneous input rather than a purely passive dose-proportional input process.

The absolute bioavailability estimates are a major strength of this study. Using the intravenous combination arm as the reference, rather than single-compound intravenous dosing, preserved the same co-administration context as the topical combination product and allowed potential systemic pharmacokinetic interactions between fluralaner and moxidectin to be incorporated into the reference exposure. In contrast, single-agent IV references would not reflect the disposition of each compound when administered together and could therefore bias the estimation of absolute bioavailability for the fixed-dose combination. The absolute bioavailability of fluralaner was 26.90%, 9.72%, and 25.88% at 40, 80, and 120 mg/kg, respectively. For moxidectin, the corresponding absolute bioavailability values were 11.74%, 10.22%, and 21.28% at 2, 4, and 6 mg/kg, respectively. These values show that only a fraction of the applied topical dose reached systemic circulation, and that this fraction varied substantially across dose levels. Topical AUC alone cannot distinguish incomplete absorption from altered systemic disposition, whereas an intravenous reference allows the extent of absorption to be estimated more directly. Therefore, the present study demonstrates that the nonlinear dose-AUC relationship was mainly attributable to dose-dependent changes in topical absorption. Together with the relatively stable terminal slopes after topical dosing, these bioavailability estimates support a contribution of variable topical systemic input to the dose–exposure pattern.

The biphasic exposure pattern may reflect a dose-dependent interplay among formulation release, local drug activity, and stratum corneum barrier function. At the medium dose, the unexpectedly lower apparent bioavailability may indicate incomplete release from the vehicle, less favorable partitioning into the stratum corneum, or reduced local thermodynamic activity. At the high dose, the supraproportional increase in C_max_ and AUC suggests that enhanced permeation predominated. Increased thermodynamic activity can increase the driving force for drug partitioning from the formulation into the skin [35]. Chemical enhancers and vehicle components may also alter lipid-domain organization in the stratum corneum and increase permeability [38]. Nanostructured and vesicular delivery systems can further modify skin partitioning and reservoir behavior [39]. In cats, additional variability may arise from hair-coat distribution, grooming behavior, and regional differences in skin physiology. Feline grooming is a structured and frequent behavior that can redistribute or remove material from the hair coat and skin surface [40,41]. Regional differences in percutaneous absorption across cat skin and variation in feline skin biophysical parameters have also been reported [42,43]. Although the present study did not directly measure drug concentrations in the skin, the plasma pharmacokinetic pattern is consistent with a cutaneous absorption process that changes qualitatively across dose levels.

The power-model analysis further confirmed that dose proportionality was not established for either analyte. The confidence interval approach based on a power model is widely used to evaluate dose proportionality in pharmacokinetic studies [22]. Practical criteria for interpreting dose proportionality have also been developed to avoid relying only on visual inspection of dose-normalized exposure [23]. In the present study, the 95% confidence intervals for the dose–exposure exponent exceeded the predefined acceptance interval for both fluralaner and moxidectin. The limited number of dose levels and the sample size of eight cats per topical group reduced the precision of the slope estimates and contributed to the width of the confidence intervals. Formal sample-size approaches for power-model dose-proportionality studies emphasize that variability, dose range, number of dose levels, and sample size jointly influence the ability to establish proportionality [44]. In practical terms, doubling the topical dose should not be assumed to double systemic exposure. Similarly, a 50% increase in nominal dose should not be assumed to produce a 50% increase in AUC or C_max_.

The absence of established dose proportionality has implications for clinical extrapolation of the fixed-combination formulation. In the dose range tested here, fluralaner was administered at 40–120 mg/kg and moxidectin at 2–6 mg/kg. Because exposure did not increase proportionally across this range, clinical exposure at intermediate or higher doses should not be inferred by assuming linearity. This is particularly important for topical products because the administered mg/kg dose, local application volume, vehicle spreading, and skin condition may all affect systemic input [34]. It is also important for fixed-combination products because both active ingredients share the same application site and vehicle, even though their systemic exposure responses may differ.

The findings are also relevant to commercial body-weight band dosing. Bravecto Plus is administered as a whole tube according to body-weight ranges rather than as an individually titrated mg/kg dose. The product label states that each tube is formulated to provide a minimum dose of 40 mg/kg fluralaner and 2 mg/kg moxidectin [45]. Because each body-weight band uses a fixed tube strength, cats near the lower end of a band will receive a higher mg/kg dose than cats near the upper end of the same band. For example, the labeled U.S. dose bands include 112.5 mg fluralaner plus 5.6 mg moxidectin for cats weighing 2.6–6.2 lb, 250 mg plus 12.5 mg for cats weighing > 6.2–13.8 lb, and 500 mg plus 25 mg for cats weighing > 13.8–27.5 lb [45]. This dosing structure means that some cats may receive doses approaching or exceeding 1.5 times the minimum recommended mg/kg dose, depending on their position within the weight band. The present data indicate that such differences in nominal mg/kg dose may not translate into proportional differences in systemic exposure. This principle is also relevant to accidental supralabel dosing, where systemic concentrations may be less predictable than expected from dose alone.

Within the experimental dose range of 40–120 mg/kg fluralaner and 2–6 mg/kg moxidectin, no adverse reactions were observed in the experimental cats. This observation supports the tolerability of the formulation under the controlled conditions of the present pharmacokinetic study. Previous field and experimental studies have also reported that fluralaner–moxidectin topical treatment was generally well tolerated in cats [31]. The safety observations in the present study represent clinical tolerability findings within a pharmacokinetic study population. Safety assessment was not the primary objective, and the study was not powered as a target-animal safety trial. Repeated dosing, diseased cats, pregnant or lactating cats, cats with neurological disorders, and cats with clinically abnormal skin at the application site were not evaluated. Product labeling also recommends caution when using isoxazoline-containing products in cats with a history of neurological disorders [45].

The highest topical dose evaluated in this study, 120 mg/kg fluralaner plus 6 mg/kg moxidectin, exceeded the maximum labeled dose range described for routine commercial dosing. The product label reports a maximum labeled dose of approximately 93 mg/kg fluralaner and 4.7 mg/kg moxidectin in margin-of-safety studies [45]. Therefore, the high-dose group in the present study should be interpreted as an exploratory supralabel pharmacokinetic condition rather than as a routine clinical dose. Including this group was nevertheless scientifically valuable because it revealed a supraproportional increase in systemic exposure that would not have been apparent from a single-dose study. From a formulation-development perspective, this finding indicates that dose escalation studies can uncover absorption behavior that may be missed when only the recommended dose is evaluated. This is especially relevant for generic or reformulated topical products because changes in solvent system, excipient composition, viscosity, spreading behavior, or drying characteristics may alter skin partitioning, local depot formation, or thermodynamic activity even when the nominal active-ingredient dose is unchanged [19,38,46].

These findings do not challenge the established efficacy of fluralaner–moxidectin products at labeled doses. Published field studies support the effectiveness of the combination against fleas, ticks, and gastrointestinal nematodes in cats [30]. Experimental studies support its activity against ear mites and feline lungworm-related endpoints [32]. A 2024 study further supported preventive efficacy against feline *Aelurostrongylus abstrusus* infection using a multi-diagnostic approach [33]. Rather, the present results refine the pharmacokinetic interpretation of such products by showing that sustained exposure and dose proportionality are distinct properties. A formulation can provide long-lasting systemic concentrations while showing non-proportional exposure across dose levels. Therefore, when evaluating new formulations, alternative vehicles, accidental supralabel exposure, or broader dosing strategies, exposure should be measured directly rather than inferred from nominal dose alone.

Several limitations should be considered. First, this was a pharmacokinetic study rather than an efficacy or target-animal safety study, so no direct exposure–response or exposure–toxicity relationship can be concluded. Second, only healthy cats were enrolled; diseased cats, cats with altered skin barrier function, pregnant or lactating cats, and cats receiving concomitant medications were not included. Third, repeated administration was not evaluated, so accumulation, changes in absorption after repeated dosing, and long-term tolerability were not assessed. Fourth, the proposed absorption-related mechanism is based on plasma pharmacokinetic profiles and established principles of topical and transdermal delivery rather than on direct dermatokinetic measurements. Skin concentrations, stratum corneum partitioning, local depot formation, penetration kinetics, and barrier integrity were not measured. Tape stripping, skin biopsy, dermal microdialysis, or in vitro permeation testing would help determine whether the observed exposure pattern is caused by altered stratum corneum partitioning, vehicle saturation, local depot formation, or a combination of these mechanisms. Fifth, each topical dose group contained eight cats and only three dose levels were evaluated, which limited the precision of the dose-proportionality analysis. Finally, grooming behavior, formulation spreading, application-site condition, plasma protein binding, tissue distribution, and excretion were not quantitatively evaluated.

In conclusion, this study demonstrates that topical administration of a fluralaner–moxidectin fixed-combination formulation produces sustained systemic exposure in cats. Dose proportionality was not established across the investigated dose range. The intravenous combination reference enabled estimation of absolute bioavailability and supported a contribution of topical systemic input to the observed exposure pattern, while formulation distribution, grooming behavior, local skin physiology, inter-individual variability, and disposition-level interaction may also contribute. The intravenous comparison also suggested a modest interaction between the two active ingredients when administered in combination. No adverse reactions were observed in experimental cats at 40–120 mg/kg fluralaner and 2–6 mg/kg moxidectin, but this tolerability finding should be interpreted within the limitations of a pharmacokinetic study. Overall, these results indicate that systemic exposure after body-weight band dosing, accidental supralabel dosing, or formulation changes should not be extrapolated by simple linear scaling, and they support direct pharmacokinetic evaluation across clinically relevant and supralabel dose ranges during development of long-acting feline topical parasiticide formulations.

## 5. Conclusions

In this study, the pharmacokinetics of a fixed-combination fluralaner–moxidectin spot-on formulation were characterized in cats across three escalating topical doses. Within the tested range of 40–120 mg/kg fluralaner and 2–6 mg/kg moxidectin, dose proportionality of fluralaner and moxidectin could not be established within the evaluated dose range and statistical power of the present study. The wide confidence intervals around the slope estimates indicate substantial variability and limited precision; therefore, the present data should not be interpreted as definitive evidence of nonlinear pharmacokinetics. The prolonged T_max_ and mean residence time, together with dose-independent terminal half-lives, indicate that the observed nonlinearity was primarily absorption-driven and was consistent with sustained cutaneous input rather than nonlinear systemic elimination. The intravenous comparison further suggested a moderate pharmacokinetic interaction between fluralaner and moxidectin when administered in combination. No adverse reactions were observed under the conditions of this study. These findings indicate that systemic exposure from this topical fixed combination should not be extrapolated by simple linear dose scaling, particularly when considering body-weight band dosing or formulation modification. Overall, the results provide a pharmacokinetic basis for dose optimization and further development of long-acting feline antiparasitic spot-on formulations.

## Figures and Tables

**Table 1:** Experimental groups and treatments.

Group	Number of Cats	Route of Administration	Drug (s)	Dose (mg/kg BW.)
A	4♂/4♀	Intravenous	Fluralaner + Moxidectin (combination)	Fluralaner: 12.5; Moxidectin: 0.5
B	4♂/4♀	Intravenous	Fluralaner alone	Fluralaner: 12.5
C	4♂/4♀	Intravenous	Moxidectin alone	Moxidectin: 0.5
D	4♂/4♀	Transdermal (spot-on)	Fluralaner + Moxidectin (combination)	Fluralaner: 40; Moxidectin: 2
E	4♂/4♀	Transdermal (spot-on)	Fluralaner + Moxidectin (combination)	Fluralaner: 80; Moxidectin: 4
F	4♂/4♀	Transdermal (spot-on)	Fluralaner + Moxidectin (combination)	Fluralaner: 120; Moxidectin: 6

**Table 2:** Key pharmacokinetic parameters of fluralaner and moxidectin following single intravenous administration (SP) or combined administration (CP).

Parameter	Units	IV-CP-Fluralaner	IV-SP-Fluralaner	IV-CP-Moxidection	IV-SP-Moxidection
AUC%Extrap	%	0.26 ± 0.07 ^a^	0.19 ± 0.04 ^b^	2.30 ± 1.1 ^3A^	2.34 ± 0.99 ^A^
AUC_0−∞_	d·ng/mL	77,727.991 ± 10,545.18 ^a^	65,412.15 ± 8723.87 ^b^	1162.46 ± 302.06 ^A^	1622.82 ± 441.93 ^B^
AUC_0−t_	d·ng/mL	77,527.95 ± 10,539.80 ^a^	65,292.74 ± 8726.43 ^b^	1137.92 ± 302.35 ^A^	1584.55 ± 428.54 ^B^
t_1/2_	d	14.99 ± 1.32 ^a^	11.86 ± 1.19 ^b^	25.60 ± 6.38 ^A^	31.02 ± 8.44 ^A^
λz	1/d	0.05 ± 0.01 ^a^	0.06 ± 0.01 ^b^	0.03 ± 0.01 ^A^	0.02 ± 0.01 ^A^
MRT_last_	d	19.94 ± 2.56 ^a^	14.60 ± 2.24 ^b^	26.29 ± 8.43 ^A^	36.23 ± 7.99 ^B^
CL	mL/d/kg	163.48 ± 22.62 ^a^	193.96 ± 24.81 ^b^	472.58 ± 196.42 ^A^	332.18 ± 105.80 ^A^
Vz	mL/kg	3524.97 ± 519.07 ^a^	3339.60 ± 635.53 ^a^	17,499.73 ± 8140.55 ^A^	14,480.62 ± 4465.34 ^A^

Note: AUC%Extrap, percentage of AUC_0−∞_ attributable to extrapolation from the last measurable time point to infinity; AUC_0−∞_, area under the concentration–time curve from 0 to infinity; AUC_0−t_, area under the concentration–time curve from 0 to the last measurable point; CL, total body clearance after intravenous administration; t_1/2_, elimination half-life; λz, elimination rate constant; MRT_last_, mean residence time from zero to the last measurable concentration; Vz, apparent volume of distribution during the terminal elimination phase. Note: For fluralaner, mean values sharing the same lowercase superscript letters do not differ significantly between single and combined administration (*p* > 0.05), whereas different lowercase letters indicate a significant difference (*p* < 0.05). For moxidectin, the same applies using uppercase letters.

**Table 3:** Key Pharmacokinetic Parameters After Intravenous Dosing of Fluralaner–Moxidectin Combination and Single Agents: Statistical Comparison.

Parameter	Units	Fluralaner-*P*	Point Estimate	Fluralaner-Lower CI 95%	Fluralaner-Upper CI 95%	Moxidectin-*P*	Point Estimate	Moxidectin-Lower CI 95%	Moxidectin-Upper CI 95%
AUC%Extrap	%	0.023	1.281	0.276	2.349	0.949	−0.330	−1.012	0.948
AUC0-∞	d·ng/mL	0.023	1.273	0.169	2.340	0.029	−1.216	−2.275	−0.122
AUC_0−t_	d·ng/mL	0.024	1.264	0.262	2.330	0.030	−1.204	−2.262	−0.112
t_1/2_	d	0.000	2.487	1.124	3.802	0.169	−0.724	−1.728	0.303
λz	1/d	0.001	−1.994	−3.195	−0.749	0.172	0.720	−0.307	1.723
MRT_last_	d	0.002	2.219	0.922	3.470	0.030	−1.211	−2.269	−0.117
CL	mL/d/kg	0.022	−1.284	−2.352	−0.178	0.097	0.890	−0.157	1.909
Vz	mL/kg	0.533	0.319	−0.673	1.301	0.373	4.600	−0.542	1.446

Note: A *p*-value of <0.05 was considered statistically significant.

**Table 4:** Key pharmacokinetic parameters of fluralaner in cats following topical application at different multiples of the recommended dose.

Parameter	Units	CP-TD-Fluralaner-40 mg/kg	CP-TD-Fluralaner-80 mg/kg	CP-TD-Fluralaner-120 mg/kg
AUC%Extrap	%	0.96 ± 0.50	0.32 ± 0.05	0.38 ± 0.35
AUC_0−∞_	d·ng/mL	67,321.89 ± 27,018.74	48,386.06 ± 18,965.39	193,394.93 ± 39,850.69
AUC_0−t_	d·ng/mL	66,739.99 ± 26,844.99	48,235.34 ± 18,913.22	192,651.15 ± 39,748.55
C_max_	ng/mL	1287.01 ± 353.03	1778.62 ± 753.92	4504.41 ± 602.58
T_max_	d	16.88 ± 6.81	10.88 ± 3.09	18.25 ± 6.32
t_1/2_	d	20.25 ± 3.70	14.17 ± 2.90	18.04 ± 3.28
λz	1/d	0.04 ± 0.01	0.05 ± 0.01	0.04 ± 0.01
MRT_last_	d	40.11 ± 6.62	25.18 ± 2.16	37.63 ± 7.49

Abbreviations: AUC%Extrap, percentage of AUC_0−∞_ attributable to extrapolation from the last measurable time point to infinity; AUC_0−∞_, area under the concentration–time curve from 0 to infinity; AUC_0−t_, area under the concentration–time curve from 0 to the last measurable point; C_max_, maximum whole-blood concentration; t_1/2_, elimination half-life; λz, elimination rate constant; MRT_last_, mean residence time from zero to the last measurable concentration; T_max_, time to maximum concentration. The same abbreviations are used hereinafter. Similarly hereinafter.

**Table 5:** Key pharmacokinetic parameters of moxidectin in cats following topical application at different multiples of the recommended dose.

Parameter	Units	CP-TD-Moxidectin-2 mg/kg	CP-TD-Moxidectin-4 mg/kg	CP-TD-Moxidectin-6 mg/kg
AUC%Extrap	%	4.51 ± 1.88	6.69 ± 8.12	2.63 ± 1.81
AUC0-∞	d·ng/mL	556.39 ± 243.39	968.67 ± 453.93	2965.34 ± 2097.52
AUC0-t	d·ng/mL	534.30 ± 242.69	930.02 ± 460.55	2906.08 ± 2099.25
Cmax	ng/mL	13.52 ± 6.53	25.10 ± 15.13	55.17 ± 21.33
Tmax	d	15.63 ± 15.47	10.50 ± 3.38	13.75 ± 8.63
t1/2	d	23.11 ± 7.75	25.85 ± 8.32	27.53 ± 8.01
λz	1/d	0.03 ± 0.02	0.03 ± 0.01	0.03 ± 0.01
MRTlast	d	36.50 ± 7.19	35.26 ± 5.28	44.68 ± 12.40

## Data Availability

The data presented in this study are available on request from the corresponding author due to the confidentiality of the project.
